# Uric acidas a predictor of bipolar disorder type 1

**DOI:** 10.1192/j.eurpsy.2021.1663

**Published:** 2021-08-13

**Authors:** Ö. Baş Uluyol, Ö. Şahmelikoğlu Onur

**Affiliations:** 1 Psychiatry, Sancaktepe Şehit Prof. Dr. İlhan Varank Research & Training Hospital, İstanbul, Turkey; 2 Psychiatry, Bakirkoy Research & Training Hospital for Psychiatry, Neurology and Neurosurgery, İstanbul, Turkey

**Keywords:** uric acid, purinergic system dysfunction, bipolar disorder

## Abstract

**Introduction:**

It has been shown in the studies that purinergic system dysfunction might have an effect on pathophysiology of bipolar disorder.

**Objectives:**

In this study, our aims were to compare uric acid levels between patient with bipolar disorder type 1 (BPD1) and healthy controls (HC) and to evaluate the validity of uric acid in predicting bipolar disorder.

**Methods:**

Consecutive outpatients with a diagnosis of euthymic BPD-1 (n=75) and HC (n=75) were included in the study. The subjects were evaluated with Sociodemographic Data Form, Young Mani Rating Scale (YMRS), Beck Depression Scale. Serum uric acid was measured using an auto analyzer.

**Results:**

Serum uric acid level in BPD1 group was significantly higher than HC. Significant efficacy of uric acid value was observed in the differentiation of BPD1 and HC [Area under the curve 0.708(0.626-0.790)]. Significant efficacy of uric acid 5.4 cut off value was observed in the differentiation of BPD1 and HC [Area under the curve 0.667 (0.579-0.8754)]. Sensitivity was 50.7%, positive predictive value (PPV) was 74.5%, specificity was 82.7%, negative predictive value (NPV) was 62.6%.

**Conclusions:**

Serum uric acid value is effective in detecting bipolar patients.

**Disclosure:**

No significant relationships.

